# Implementation research to support Bangladesh Ministry of Health and Family Welfare to implement its national guidelines for management of infections in young infants in two rural districts

**DOI:** 10.1186/s41043-019-0200-6

**Published:** 2019-12-06

**Authors:** Salahuddin Ahmed, Jennifer A. Applegate, Dipak K. Mitra, Jennifer A. Callaghan-Koru, Mahfuza Mousumi, Ahad Mahmud Khan, Taufique Joarder, Meagan Harrison, Sabbir Ahmed, Nazma Begum, Abdul Quaiyum, Joby George, Abdullah H. Baqui

**Affiliations:** 1 Johns Hopkins University-Bangladesh, Dhaka, 1213 Bangladesh; 2 International Center for Maternal and Newborn Health, Department of International Health, Johns Hopkins Bloomberg School of Public Health, Baltimore, MD 21205 USA; 3 Department of Public Health, School of Health and Life Sciences, North South University, Dhaka, 1229 Bangladesh; 4 Department of Sociology, Anthropology, and Health Administration and Policy, University of Maryland, Baltimore County, Baltimore, MD USA; 5 Jhpiego, Baltimore, MD USA; 6 BRAC James P Grant School of Public Health, BRAC University, Dhaka, Bangladesh; 7 USAID’s MaMoni Health Systems Strengthening Project, Save the Children, Washington, DC USA; 8 Maternal and Child Health Division, icddr,b, Dhaka, 1212 Bangladesh

**Keywords:** Implementation research, Young infant infection, Possible serious bacterial infection, Outpatient management; Bangladesh

## Abstract

**Background:**

World Health Organization revised the global guidelines for management of possible serious bacterial infection (PSBI) in young infants to recommend the use of simplified antibiotic therapy in settings where access to hospital care is not possible. The Bangladesh Ministry of Health and Family Welfare (MoHFW), Government of Bangladesh (GOB) adopted these guidelines, allowing treatment at first-level facilities. During the first year of implementation, the Projahnmo Study Group and USAID/MaMoni Health Systems Strengthening (HSS) Project supported the MoHFW to operationalize the new guidelines and conducted an implementation research study in selected districts to assess challenges and identify solutions to facilitate scale-up across the country.

**Implementation support:**

Projahnmo and MaMoni HSS teams supported implementation in three areas: building capacity, strengthening service delivery, and mobilizing communities. Capacity building focused on training paramedics to conduct outpatient management of PSBI cases and developing monitoring and supervision systems. The teams also filled gaps in government supply of essential drugs, equipment, and logistics. Community mobilization strategies to promote care-seeking and referrals to facilities varied across districts; in one district community, health workers made home visits while in another district, the promotion was carried out through community volunteers, village doctors, and through existing community structures.

**Methods:**

We followed a plan-do-study-act (PDSA) cycle to identify and address implementation challenges. Three cycles—1 every 4 months—were conducted. We collected data utilizing quantitative and qualitative methods in both the community and facilities. The total sample size for this study was 13,590.

**Discussion:**

This article provides implementation research design details for program managers intending to implement new guidelines on management of young infant infections. Results of this research will be reported in the forthcoming papers. Preliminary findings indicate that the management of PSBI cases at the UH&FWCs is feasible. However, MoHFW, GOB needs to address the implementation challenges before scale-up of this policy to the national level.

## Background

Global rates of child mortality have dropped significantly over the past few decades, with remarkable declines seen for older children. However, mortality rates in neonates remain high with an estimated 2.7 million annual deaths globally [1]. About 45% of all deaths in children under 5 years of age occur during the neonatal period [2] and almost 98% of neonatal deaths occur in developing countries [1]. Globally, an estimated one-fourth of the neonatal deaths are attributed to infectious causes, and in settings characterized by high neonatal mortality rates, the proportion of neonatal deaths due to infections is estimated to be even higher [3, 4]. In Bangladesh, newborn infections remain a major cause of both morbidity and mortality [4, 5]. About 37% of all neonatal deaths in Bangladesh occur as a result of sepsis or other severe infections [6].

The World Health Organization (WHO) recommends that young infants (0-59 days) with signs of possible serious bacterial infection (PSBI) be referred to hospitals for treatment with a 7–10 day course of two injectable antibiotics–penicillin (or ampicillin) and gentamicin. However, referral compliance for hospitalization in many developing countries has been low due to limited access or inadequate hospital facilities [7, 8]. In 2007, the WHO, United States Agency for International Development (USAID), and Save the Children’s Saving Newborn Lives program (SC/SNL) convened an expert panel aimed at identifying simple, safe, and effective treatment regimens that could be provided to young infants with severe infections closer to home when the family was not able to accept referral to the hospital [9]. The panel concluded that the existing evidence was insufficient to recommend antibiotic treatment for severe infections at the community level and identified the need for additional research on the efficacy of simplified antibiotic therapy [9]. Three randomized, open-label, equivalence trials were conducted in Bangladesh, Pakistan and three countries in Africa (Democratic Republic of Congo [DRC], Kenya, and Nigeria) to evaluate the efficacy of simplified antibiotic regimens for managing PSBI in young infants at the community level when referral was not possible [10]. While the trial protocols were harmonized, the number of doses and service delivery mechanisms varied across the studies. Findings from all three studies demonstrated that the simplified regimens were as efficacious as the standard regimen [11–13].

In 2015, the WHO revised the global guidelines recommending use of simplified antibiotic regimens for the management of PSBI in young infants for resource-limited settings when hospitalization is not acceptable or accessible to families [14]. The Government of Bangladesh (GOB) adopted the WHO guidelines and developed a corresponding policy, titled *Management of Infection of the 0–59 Days Infants at Union Level Facilities and NGO Clinics without Indoor Facilities* [15]. The union level facilities under the management of the Ministry of Health and Family Welfare (MoHFW) in rural Bangladesh are known as health and family welfare centers (UH&FWCs). In most administrative unions, there is one UH&FWC, which serves a catchment population of approximately 25,000 persons [16, 17]. The UH&FWC provides mostly outpatient services. The services offered at the UH&FWCs include essential maternal, newborn, child health, family planning, and nutrition services, including the management of normal vaginal deliveries. It is staffed with one Sub-Assistant Community Medical Officer (SACMO) who has at least 3 years of training on general health care including child health and at least one Family Welfare Visitor (FWV) who has at least 18 months of training on pregnancy care and family planning.

The Comprehensive Newborn Care Package (CNCP) was developed for the implementation of newly recommended priority newborn interventions, including management of infections in young infants. With implementation of the new guidelines, SACMOs are being trained with CNCP to assess and treat infants with PSBI. Per the updated guidelines, the SACMO assesses the infant and determines an illness classification based on the standardized Integrated Management of Childhood Illness (IMCI) algorithm for infants under 2 months of age (Table 1).

**Table 1:** Operational algorithm for managing infections in young infants in UH&FWC per the Bangladesh guidelines

Category	Clinical signs	Management	Follow-up and referral support
Critical illnesses (CI)	• Unconscious/drowsy • Convulsion/history of convulsion • Unable to feed • Persistent vomiting • Central cyanosis • Bulging fontanel • Weight < 1500 g	• The young infant will be administered the 1st dose of injectable gentamicin and oral antibiotic (if possible), advised about the importance of hospitalization and referred urgently to the designated referral facility with a referral slip containing referral notes of SACMO • The mother will be advised on frequent breastfeeding to prevent low blood sugar. She will also be properly advised to keep the baby warm especially during transportation.	• The mobile phone contact number will be kept to follow-up the referral compliance. The phone number of the SACMO will be provided to the family • The UH&FWC service providers will communicate with the Upazila Health Complex (UHC) (referral center) about the case • Necessary support to be provided by UH&FWC service providers or field supervisors to arrange transport for referral
Clinical severe infection (CSI)*	• Severe chest in-drawing • Hypothermia (< 95.9 ^°^F or 35.5 ^°^C • Raised temperature (> 99.5 ^°^F or 37.5 ^°^C) • Less movement/movement only when stimulated • Not feeding well (depending on history and observation)	• The case will be administered 1st dose injectable gentamicin and oral amoxicillin, and referred following the above procedure to the nearest UHC for management	• Same as above, the SACMO’s mobile number will be given to caregiver and the case will be followed up over phone to record referral compliance on the day of referral by UH&FWC provider
		In case of referral non-compliance: • The infant will be managed by the SACMO using standard management protocol: o Injection gentamicin I/M once daily at UH&FWC for 2 days o Oral amoxicillin twice daily for 7 days • The family will be counseled and advised to come to the same facility with the baby to receive the 2nd (last) dose of injectable antibiotic and continue oral medicine 12 hourly for total 7 days	• On the 2nd day of treatment, the infant should return to UH&FWC for assessment and 2nd dose injectable gentamicin • On the 4th and 8th day of treatment, follow-up will be conducted to assess condition of the infant • If the baby develops any new symptom (listed symptoms of CSI or CI), does not improve after 4 days of receiving treatment or, is not fully cured after treatment completion (on the 8th day); the family should be advised for immediate notification to the same service provider and to seek care from referral facility
Isolated fast-breathing as single sign of illness	• Young infants 0–6 days old with fast breathing as the only sign of illness*	• Give 1st dose of oral amoxicillin and refer to UHC	• The mobile phone contact number will be kept to follow-up the referral compliance. The phone number of the SACMO will be provided to the family • The UH&FWC service providers will communicate with the UHC (referral center) about the case
		In case of referral non-compliance: • The infant will be managed by the SACMO using standard management protocol: o Oral amoxicillin (100 mg/kg/day twice daily) for 7 days	• Infant will be followed up on the 4th day and 8th day • If the baby develops any new symptom (listed symptoms of CSI or CI) or, does not improve after 4 days of receiving treatment, or is not fully cured after treatment completion (on 8th day), the family should be advised for immediate notification to the same service provider and to seek care from referral facility
	• Young infants 7–59 days old with fast breathing as the only sign of illness	• No referral, treated with oral amoxicillin (100 mg/kg/day twice daily) for 7 days	• Sick infants with fast-breathing (7–59 days) will be followed up on the 4th day and 8th day
Local bacterial infection	• Umbilical redness • Draining pus from umbilicus • Skin pustule	• No referral, treated with oral amoxicillin (125 mg daily for below 1-month aged infants or infants having less than 4 kg weight and 250 mg for infants aged between 1 and 2 months) for 5 days	• Caregiver will be advised to seek immediate consultation with UH&FWC provider if infant does not improve, new symptoms appear, or condition worsens

*PSBI cases eligible for simplified antibiotic treatment when hospital referral is not feasible for families

If the SACMO identifies any signs of PSBI, then the SACMO is trained to administer the first dose of injectable and/or oral antibiotics and refer the infant to the Upazila (sub-district) Health Complex (UHC). If the family declines referral to the hospital, then the SACMO either reinforces referral or treats the infant depending upon classification per the guidelines, which also includes providing medicine to be administered at home by the caregiver. The ability of the SACMO to treat infants with non-critical illnesses on an outpatient basis is the primary change to the previously established treatment protocol. According to the protocol, the SACMO also follows-up PSBI cases at day 4 through phone or in the facility if the parent brings the infant for a follow-up visit. During follow up, the SACMO decides whether to continue treatment (if condition improved) or refer to the higher facility for further management (if condition has not improved or new symptoms developed).

The other cadres of providers involved in outpatient management of PSBI cases are the FWV and Family Planning Inspectors (FPI). The FWVs are posted at the UH&FWC and primarily provide antenatal care, normal delivery care, postnatal care and family planning services to the community. FWVs are able to provide the second dose of injectable gentamicin to PSBI cases in the absence of the Sub-Assistant Community Medical Officers (SACMO) [15]. FPIs are non-clinical field supervisors of frontline workers in the community. For PSBI management, the FPIs are trained and engaged for follow-up of the infant at the end of treatment (day 8 follow-up) within the community. During these home visits, FPIs assess the condition of the infant, record any existing signs or symptoms, determine the condition of the infant (i.e., recovered or not recovered), and advise on referral if the infant has not recovered.

Prior to national scale-up of the guidelines, the Bangladesh MoHFW planned to learn from implementation of the policy in three selected districts of Bangladesh: Kushtia, Lakshmipur, and Sylhet. We conducted an implementation research study in the first year of this program (September 2015–August 2016) to document the inputs and processes required for operationalization of the updated policy in varying contexts, identify barriers and facilitators for implementation, and integrate these early lessons into the plans for national scale-up. This paper describes the implementation research protocol followed by the Projahnmo and MaMoni Health System Strengthening (HSS) teams who provided support to the MoHFW in Sylhet and Lakshmipur, respectively. A third partner provided support in Kushtia, but their methodology is not described in this paper.

## Methods

### Study setting

**Fig. 1:**
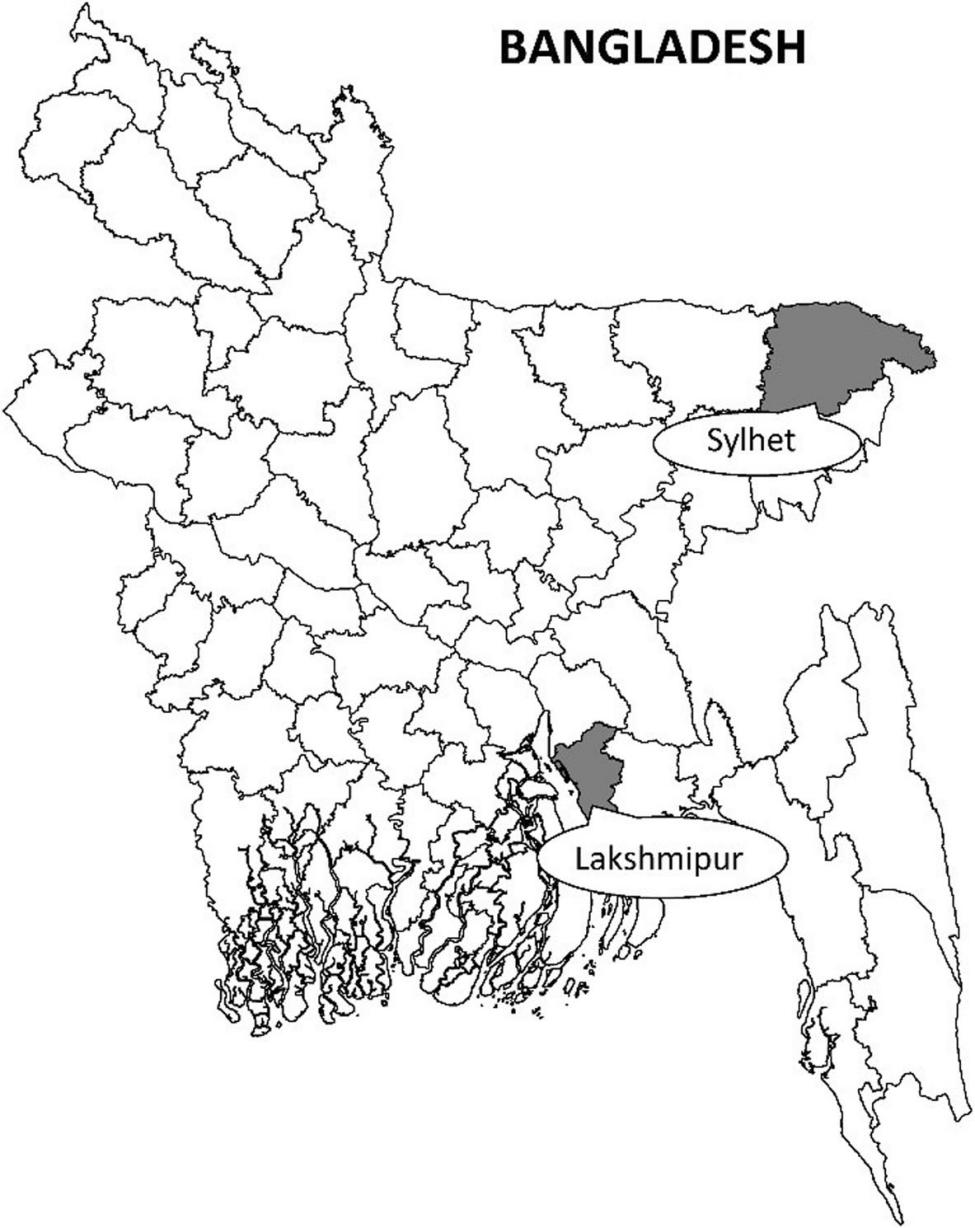
Map of Bangladesh highlighting implementation research study area districts

This implementation research was conducted in two sub-districts of Sylhet district in Sylhet division and one sub-district of Lakshmipur district in Chittagong division (Fig. 1). Sylhet and Chittagong are historically low performing divisions of Bangladesh for maternal, newborn, and child health indicators. According to the 2014 Bangladesh Demographic and Health Survey, mothers in Sylhet had the lowest proportion of births in facilities (22.6%) and lowest proportion of births attended by a skilled provider (27.1%) [6], followed by Chittagong division where 35.2% deliveries took place in facilities, and 43.9% of the deliveries were attended by a medically trained provider [6].

### Study design

During the first year of implementation of the updated PSBI guidelines, the MoHFW received implementation support from Projahnmo and MaMoni HSS project in the selected districts, Sylhet and Lakshmipur, respectively. Projahnmo is a partnership of the Johns Hopkins University with the Bangladesh MoHFW and Bangladeshi NGOs. Projahnmo has been working in Sylhet since 2001 and has extensive experience with designing and evaluating newborn and maternal health interventions [18]. Projahnmo provided support to the implementation of the PSBI guidelines in two sub-districts of Sylhet: Zakigonj and Kanaighat. The USAID-funded MaMoni HSS project is implemented in six districts of Bangladesh with the goal of improving utilization of integrated maternal, newborn, child health, family planning, and nutritional services [19]. The project inputs are primarily focused on improving the performance and capacity of health services at the district level. Since 2003, MaMoni HSS project has been working in all upazilas in Lakshmipur to strengthen district-level health systems and promote scale-up of maternal, neonatal and child health, family planning, and nutrition (MNCHFPN) interventions [19]. For this study, MaMoni HSS provided support to the implementation of the PSBI guidelines in one sub-district of Lakshmipur (Ramgonj).

Both Projahnmo and MaMoni HSS partnered with the MoHFW to facilitate program trainings, ensure drug availability, and conducted joint supervision visits with the MoHFW to the first-level facilities targeted for implementation. The measurement and evaluation component of this study was led by Projahnmo, with support of MaMoni HSS in Lakshmipur, utilizing a mixed-methods approach to assess the following implementation research objectives:

1. Examine feasibility of implementation of the newly developed infection management guidelines in young infants at UH&FWCs through outpatient services when referral is not accepted
2. Assess the acceptability of infection management services delivered on an outpatient basis at UH&FWCs among the parents and families of young infants
3. Measure caregiver’s knowledge and coverage of infection management for young infants
4. Assess the compliance of the families to the referral advice and new treatment regimen for young infant infections delivered at UH&FWCs
5. Document the safety of the injectable antibiotic therapies delivered at union level facilities as per national guidelines for infants classified as clinical severe infection who refuse referral advice
6. Identify barriers and facilitating factors to the implementation of the protocol, and develop strategies to address barriers to be incorporated into national scale-up plans

### Implementation support to the MoHFW

#### Training on the guidelines for outpatient management of young infants with PSBI

In coordination with the MoHFW and Bangabandhu Sheikh Mujib Medical University (BSMMU), Projahnmo and MaMoni HSS facilitated a training-of-trainers for district- and upazila-level service providers (e.g., SACMO, FWV, FPI). Additionally, implementation support teams organized orientation of both government and program-supported health workers and volunteers within the community to promote identification of danger signs and the referral of cases identified in the community to sub-district and union-level facilities. The team also supported the training of FPIs on identification of potential infection cases, referrals, and follow-up of sick young infants in the community. Refresher trainings were provided to improve the quality of record keeping, PSBI case management, referral, and follow-up by SACMOs and FPIs.

#### Support to monitoring and supervision of UH&FWC providers

District and sub-district level MoHFW managers were responsible for routine supervision and monitoring of SACMOs and FPIs. Both Projahnmo and MaMoni HSS facilitated joint supervision visits with local MoHFW managers at UH&FWCs within the study areas. During these visits, implementation support teams joined the managers in their supervision of SACMOs to observe the quality of supervision and discuss and resolve challenges with both supervisors and providers in real-time. The support team provided on-the-job training and mentoring to SACMOs focusing on PSBI management, record keeping, and monitoring. MaMoni HSS also attended the monthly meetings for the SACMOs at the UHC in Ramgonj to support preparation of monthly reports.

#### Supply of drugs, equipment, and logistics required for PSBI management

The implementation-support teams coordinated with the MoHFW and used project’s discretionary funds to procure essential drugs, equipment, and logistics during the initial implementation period. They worked with the MoHFW to procure the necessary drugs and supplied them through government channels for an interim period while the system for supplies through the MoHFW was being worked out.

#### Care-seeking message dissemination by Projahnmo Community Health Workers (CHW) through home visits

As part of other projects ongoing in Sylhet under Projahnmo, there was an existing cadre of CHWs providing home visits to mothers, newborns, and children once in every 2 months. CHWS are local women with at least tenth grade education, who receive 6 weeks of basic health training, and each CHW serve a population of about 4000 persons. Projahnmo conducted a 1-day training in the first months of implementation to orient CHWs on the updated guidelines for management of infections in young infants. The CHWs promoted identification of danger signs in infants and disseminated the following messages: (1) when illness is identified, caregivers should take sick young infants to sub-district hospitals and (2) if they were unable to go to the hospital, they should seek care for the infant at the UH&FWC.

#### Promotion of care-seeking and referrals through Expanded Program on Immunization (EPI) and satellite sessions

Family Welfare Assistants (FWA) and Health Assistants (HA) are the government frontline health workers who conduct home visits and register pregnancies and newborns as a part of their routine responsibilities. FWAs and HAs received a 1-day training on the available services for treatment of PSBI for young infants at the UH&FWC. FWAs and HAs were trained to disseminate this message to the mothers in the community during their regular home visits, EPI, and satellite sessions.

#### Engaging community volunteers and village doctors to promote care-seeking and referrals

Community Volunteers (CV) (1 for 250 population) of MaMoni HSS project were oriented on newborn danger signs, availability of sick child management services offered at the UH&FWC, and appropriate referral. They disseminated these messages within their communities through a monthly Community Action Group meeting (CAG). In addition to awareness development, these CVs interface with community level GOB health workers (e.g., HA, FWA) at community microplanning meetings held monthly at the outreach EPI center. CVs support the MoHFW frontline health workers to gather information of births, maternal, or newborn deaths and refer sick newborns in their area. Additionally, MaMoni HSS project oriented the village doctors on identification of PSBI cases and referred them to SACMOs as they are often the first point of care for sick infants at the community level.

#### Engagement of community groups for improving care-seeking and referrals in the community

Projahnmo study staff also oriented members of community groups in Sylhet on newborn danger signs, the importance of care-seeking, and the new services available at the UH&FWC. Community groups are the local governing body for community clinics, which are the lowest tier government facility providing primary healthcare on an outpatient basis to a catchment area of about 6,000 in population [20]. The community group meets periodically to discuss the progress, challenges, and local solutions at their forum. The Projahnmo team oriented community group members to disseminate these awareness messages among mothers, caregivers, and other community members to bolster care-seeking and community referrals for sick infants.

### Implementation research methods

Over the course of the 1-year implementation research study (September 2015–August 2016), we conducted an evaluation, independent of the implementation support, which employed mixed-methods data collection activities in 19 unions located in two sub-districts of Sylhet (9 unions) and one sub-district of Lakshmipur (10 unions). A convergent parallel mixed-methods design was used to guide quantitative and qualitative data collection, analysis, and interpretation of study results. Quantitative data were collected through rolling household surveys, periodic health facility assessments, weekly extraction of data from health facility records of young infants, and continuous follow-up surveys with caregivers of infection cases in the community. Qualitative data were collected through process documentation activities, in-depth interviews with senior level program implementers, in-depth interviews (IDI), and focus group discussions (FGD) with UH&FWC service providers, and IDI and FGD with caregivers. Both quantitative and qualitative data activities were used to assess each study objective (Table 2).

**Table 2:** Data collection activities by study objective

Study objective	Data collection activities	
	Quantitative	Qualitative
Examine feasibility of implementation of the newly developed infection management guidelines in young infants at UH&FWCs through outpatient services when referral is not accepted	• Health facility assessment	• IDI & FGD with UH&FWC service providers • IDI with MoHFW program implementers • Process documentation of implementation support activities
Assess the acceptability of infection management services delivered on an outpatient basis at UH&FWCs among the parents and families of young infants	• Follow-up surveys with caregivers of infection cases in the community	• IDI with caregivers of infection cases
Measure caregiver’s knowledge and coverage of infection management for young infants	• Household survey with caregivers of young infants	• FGD with caregivers of young infants
Assess the compliance of the families to the referral advice and new treatment regimen for young infant infections delivered at UH&FWCs	• Weekly review of young infant records at UH&FWC • Follow-up surveys with caregivers of infection cases in the community	• IDI with caregivers of infection cases
Document the safety of the injectable antibiotic therapies delivered at union level facilities as per national guidelines for infants classified as clinical severe infection who refuse referral advice	• Weekly review of young infant records at UH&FWC • Follow-up surveys with caregivers of infection cases in the community	• IDI with caregivers of infection cases
Identify barriers and facilitating factors to implementation of the protocol, and develop strategies to address barriers to be incorporated into national scale-up plans	• Health facility assessment • Follow-up surveys with caregivers of infection cases in the community	• IDI & FGD with UH&FWC service providers • IDI with MoHFW program implementers • Process documentation of implementation support activities

### Quantitative Data Collection and Sample Size

The health facility checklist was developed in collaboration with study partners based on the updated Bangladesh guidelines for PSBI management, which focuses on capturing health systems data on service availability, general service readiness, and service-specific readiness [15, 21]. The evaluation team piloted the checklist in July 2015 and adapted questions prior to baseline data collection. The baseline checklist was administered prior to the government’s rollout of the guidelines in 31 selected health facilities in Sylhet and Lakshmipur. The baseline checklist assessed facility readiness to implement the new guidelines including the availability of staff, drugs, and equipment. UH&FWCs were excluded if the SACMO post was vacant at the time of the baseline health facility assessment. A total of 9 UH&FWCs were selected in Zakigonj and Kanaighat, Sylhet, and 10 UH&FWCs were selected in Ramgonj, Lakshmipur. The health facility checklist was administered at two additional time points during the study period, 4 months after the start of implementation and then at the end of the study (August 2016). Data collectors also visited the UH&FWC weekly to abstract data from facility records on the number of young infants that sought services. This activity provided utilization data including the number of young infants classified with signs of infection, frequency of follow-up, and treatment received.

Rolling household surveys were administered to explore infant illness and care-seeking history, maternal knowledge, and maternal perception of severity of danger signs. Household screening and the survey were conducted by a trained group of CHWs in the study areas from November 2015-August 2016. CHWs recruited for this study identified all recently delivered women and their live born babies (0–59 days) in the included catchment areas by visiting all households during the two monthly scheduled home visits. Only married women of reproductive age (MWRA) (13–49 years) having a live birth as a pregnancy outcome and residing in the selected unions during the study period were eligible to participate in the household survey. It took approximately 2–3 months to screen and administer the survey in all the 19 UH&FWC catchment areas. Thus, a MWRA with a young infant could only be enrolled in the survey once during the study period. The household survey questionnaire was developed utilizing questions from previous household surveys, which were administered in the Sylhet study area and published by the Projahnmo research group [18, 22, 23]. The population size in our study areas is ~ 250,000 in each of the districts with an annual birth cohort of 6250 (2.5% CBR). Based on our previous estimates, we expected that 38% of young infants will be sick as per mother’s reports for at least once in the first 2 months of their life [24]. Applying this estimate, there will be ~ 2375 cases of reported sickness in a 1-year period in each of the areas.

We estimated current rates of care-seeking for sick young infants from union health facilities to be 4% based on previous data. We required 76 sick infants in each round of the survey to estimate the increase of care-seeking from union facilities from 4% at baseline to 20% at end line with 80% power. Our primary outcome was reported sickness and care-seeking in the preceding 14 days on the day of survey. All women that delivered a live birth in the preceding 60 days of the date of survey were asked to participate in the morbidity and care-seeking surveys if caregivers were able to recall illness episodes. Applying 38% cumulative incidence of reported infant illness in the first 2 months of life, we expected 9% of caregivers to report infant illness in the 14-day recall period. Thus, we targeted 845 caregivers of young infants to identify 76 sick infants in each round of the survey. Assuming a response rate of 80%, which allows for an estimated 20% rate of refusal or caregiver absence at the time of the household visit, we targeted an estimated 1055 caregivers of young infants per round of the survey (every 2–3 months). In order to achieve this target sample size, we screened all women of reproductive age for inclusion in the survey throughout the study period.

The study team also aimed to follow-up all young infants managed under the updated guidelines to assess compliance with follow-up, treatment outcomes, and safety of the regimen. Facility utilization data were collected through weekly review of the sick infant registers at the UH&FWC by our study team to assess the number of infants classified with infection, referrals, and treatment data. The study team used these records to identify young infants for follow-up in the community. To measure treatment compliance assuming 50% compliance rate with 10% precision and accounting for 10% loss to follow-up, we required complete data from follow-up with 107 young infants treated for infection in each study area. Assuming 12% average care-seeking, we estimate that about 285 sick young infants will seek care from union level facilities (12% of 2375 expected cases). However, we aimed to follow-up all young infants diagnosed and managed under the new infection management guidelines to measure the safety of the program, which will also provide compliance data. Follow-up of sick young infants was continuous throughout the study period as the aim was to follow-up all young infant diagnosed with infection.

The total sample size requested for this study was estimated at 13,590 subjects. To obtain this sample size in the community, we obtained permission to screen 50,000 women of reproductive age in each study area totaling 100,000 women of reproductive age during the study period.

### Qualitative data collection and sampling

Qualitative data collection took place concurrently during the study period to assess program feasibility and acceptance of the guidelines among MoHFW providers, managers responsible for program implementation, and caregivers of young infants. Among providers, perceptions of PSBI treatment at first-level facilities were collected using semi-structured IDIs with SACMOs and FPIs. We also conducted FGDs with FWVs. The SACMO at each of the selected UH&FWCs was asked to participate in at least one, but no more than two IDIs during the study period. Interviewers asked SACMOs and FWVs about their experience with the guidelines, opinions on training and routine supervision, and facility functioning. IDIs with FPIs were conducted in the last round of data collection (June–August 2016) to explore challenges with follow-up of infants in the community.

A subset of caregivers was selected from the list of all caregivers with young infants identified in the study area for FGDs as part of the qualitative component of this study. We aimed to explore community perceptions of young infant illness, care-seeking behaviors for illness episodes, and perceptions of care at the UH&FWC. For FGDs, caregivers were selected through convenience sampling of mothers (13–49 years) of infants under 6 months of age who were willing and able to share their experiences with care-seeking for infant illness. The number of participants for each focus group ranged from six to eight mothers.

Caregivers of sick young infants receiving outpatient treatment for PSBI were followed up in the community to assess treatment compliance. We aimed to conduct in-depth interviews with a subset of 30 of these caregivers in each study area throughout the study period. We purposively selected caregivers for interviews based on their infant’s categorization of infection. We conducted IDIs with caregivers of infants for each category of infection (i.e., critical illness, clinical severe infection, fast breathing as a single sign, and local bacterial infection). The goal of these interviews was to assess the caregiver’s experience with outpatient treatment, and reasons for non-compliance to the prescribed treatment and follow-up visits.

### Stakeholder workshops

**Fig. 2:**
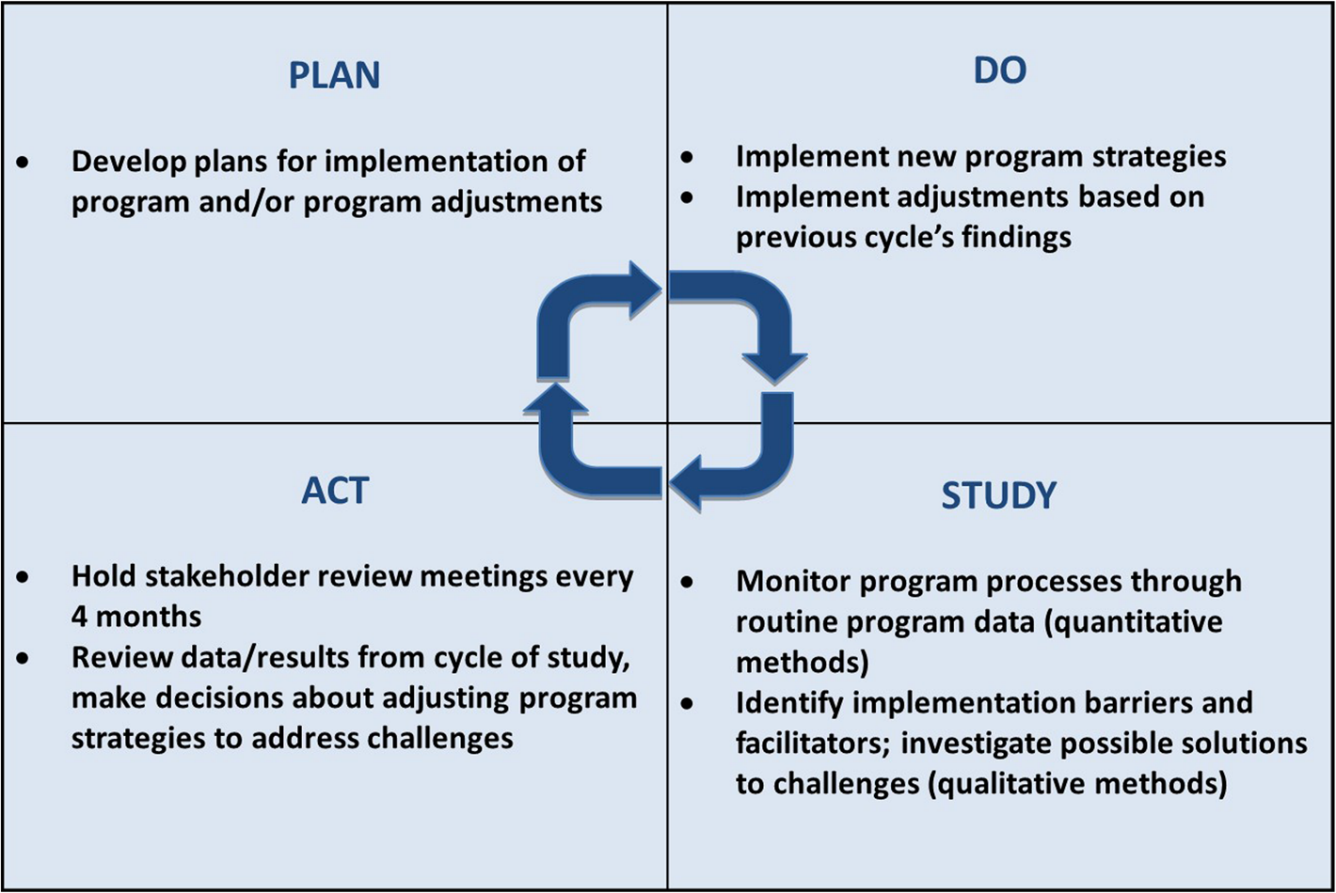
Adapted “plan-do-study-act” cycle including study activities at each stage. This implementation research study adopted an adapted action learning cycle approach, or a “plan-do-study-act” (PDSA) cycle [25, 26] to guide program learning and inform adjustments to implementation support

This implementation research study adopted an adapted action learning cycle approach, or a “plan-do-study-act” (PDSA) cycle, also known as the Deming Cycle [25, 26]. According to the PDSA approach, program implementation was studied periodically, which provided implementers with an opportunity to identify and address implementation challenges in real-time. With each cycle, data were collected on the program implementation strengths and challenges and were reviewed by a group of stakeholders. The stakeholders then developed solutions to address the challenges identified in the previous cycle and implemented these changes in the subsequent cycle (Fig. 2). The successes and challenges of the revised program approach were studied in the subsequent cycle. We arranged a stakeholders’ meeting following each round of data collection, during which the preliminary results were reviewed, and stakeholders assessed the implementation progress and challenges. The records from the stakeholder meetings served as documentation of the program learning and were reported alongside the results from data collection activities. The evaluation team worked closely with the implementation support team and the MoHFW implementers to perform all the preparatory works and organized stakeholder review meetings for sharing and gathering inputs.

A total of two stakeholder workshops were held in Dhaka after the first and third round of data collection, in January and September 2016, respectively. These workshops aimed to bring together implementation and study partners, district level officials, and stakeholders at the central level to share findings related to both successes and challenges that arose during the process of implementing the new guidelines. Through these workshops, participants shared early learnings from implementation support and evaluation activities and worked together to develop solutions to better support the implementation of the new guidelines.

### Data Analysis

Quantitative data were entered and stored in Microsoft SQL server and analyzed using Stata Special Edition 14 (College station, Texas, USA) [27]. The analysis plan for this data included summary statistics of distribution and cross-tabulation of indicators using the appropriate tests for significance (e.g., Student’s *t* test and chi-square). Qualitative data were analyzed following an adapted Framework approach [28] for identification of inductive and deductive themes. A codebook was developed to ensure consistency in the broader thematic concepts that was sought in the data. Johns Hopkins University (JHU) qualitative researchers applied thematic codes systematically to the data and examined for patterns. The interviews were transcribed and translated into English. The JHU qualitative team coded the English transcripts and analyzed as per a framework for analysis based on the objectives of the program and its implementation.

### Ethical approval

We obtained ethical clearance to conduct the study from ethical review committee and/or internal review boards of Bangladesh Institute of Child Health and Johns Hopkins Bloomberg School of Public Health. They reviewed and approved the research plan, consent forms, and data collection forms.

## Discussion

This article describes the design of an implementation research study, which included support to the Bangladesh MoHFW to implement revised guidelines for the management of young infants suffering from PSBI and a mixed-methods evaluation. The evaluation aimed to identify facilitators and barriers to the implementation of the guidelines in first-level health facilities to inform scale-up. The WHO guidelines are intended to be adopted by national governments and implemented by health workers in limited resource settings. Thus, there is a need to study how these guidelines will be implemented outside of randomized controlled trials. Implementation research provides an opportunity to understand what, why, and how interventions work in real-world conditions [29]. Our incorporation of implementation research outcomes provides the opportunity for us to assess why the program was successful or unsuccessful in meeting goals, which will be valuable feedback for both the MoHFW, WHO, USAID, and other global stakeholders [30–32]. WHO is coordinating additional implementation research studies for PSBI guideline rollout in Pakistan, India, Nigeria, Malawi, DRC, and Ethiopia. Findings from this study will be disseminated among program managers, policy-makers, development partners, and other stakeholders.

The strength of this study is the use of both quantitative and qualitative approaches to provide a deeper understanding of the research questions than either method separately [33, 34]. This approach is well-suited to the implementation research because it provides a way to understand multiple perspectives and multiple outcomes grounded within local context [29, 35].

Given the lack of a control group and short study period, it will not be possible to causally link implementation support activities to observed changes in the population. For the household survey, this limitation is exacerbated because we do not have survey data collected prior to the MoHFW’s rollout of the PSBI guidelines in the study areas. It is important to note that the lack of a comparison group and lack of randomization make the study more vulnerable to internal and external threats to validity. We aimed to improve internal validity by collecting data at multiple points in time. However, our study period was limited to 1-year, which was necessary based on the GOB’s plans for scale-up.

The potential lack of generalizability of these study findings to other developing country settings is another limitation of this study. Although generalizability was not a primary goal for this study, it will be important to consider this when formulating conclusions. This implementation research study focuses on implementation research outcomes in the Bangladesh health system, thus findings will not be directly transferable to other countries. Given that this study is being conducted prior to national scale-up of the program, it will also be important to consider the generalizability of the findings to other areas in the country. Both Sylhet and Lakshmipur have well-established, large-scale programs aimed to improve maternal, newborn and child health. As a result, it will be difficult to tease out the improvements in maternal knowledge or care-seeking that may be linked to the community mobilization activities. When formulating study conclusions, it will be important to describe the other programs operating in each area and the impact these programs may have on study findings.

## Data Availability

Not applicable

## Abbreviations

BSMMU: Bangabandhu Sheikh Mujib Medical University
CAG: Community Action Group
CBR: Crude birth rate
CHW: Community health workers
CNCP: Comprehensive Newborn Care Package
CV: Community volunteer
DRC: Democratic Republic of Congo
EPI: Expanded Program on Immunization
FGD: Focus group discussion
FPI: Family Planning Inspector
FWA: Family Welfare Assistant
FWV: Family Welfare Visitor
GOB: Government of Bangladesh
HA: Health Assistant
IDI: In-depth interviews
IMCI: Integrated Management of Childhood Illness
JHU: Johns Hopkins University
MaMoni HSS: MaMoni Health Systems Strengthening
MNCHFPN: Maternal, Neonatal and Child Health, Family Planning and Nutrition
MoHFW: Ministry of Health and Family Welfare
MWRA: Married women of reproductive age
PDSA: Plan-do-study-act cycle
PSBI: Possible serious bacterial infection
SACMO: Sub-Assistant Community Medical Officer
SC/SNL: Save the Children/Saving Newborn Lives
UH&FWC: Union Health and Family Welfare Center
UHC: Upazila Health Complex
USAID: United States Agency for International Development
WHO: World Health Organization

## References

[1] United Nations Children’s Fund (2015). Levels & trends in child mortality: report 2015. Estimates developed by the UN Inter-agency Group for Child Morality Estimation.

[2] Liu L (2016). Global, regional, and national causes of under-5 mortality in 2000-15: an updated systematic analysis with implications for the Sustainable Development Goals. Lancet.

[3] Lawn JE, Cousens S, Zupan J (2005). 4 million neonatal deaths: when? where? why?. Lancet.

[4] Liu L (2015). Global, regional, and national causes of child mortality in 2000-13, with projections to inform post-2015 priorities: an updated systematic analysis. Lancet.

[5] Baqui AH (2016). Effect of community-based newborn care on cause-specific neonatal mortality in Sylhet district, Bangladesh: findings of a cluster-randomized controlled trial. J Perinatol.

[6] BDHS, Bangladesh Demographic and Health Survey 2011. 2013, National Institute of Population Research and Training (NIPORT), Mitra and Associates, and Macro International: Dhaka, Bangladesh and Calverton, Maryland, USA.

[7] Al-Eidan FA (1999). Sequential antimicrobial therapy: treatment of severe lower respiratory tract infections in children. J Antimicrob Chemother.

[8] Peterson S (2004). Coping with paediatric referral--Ugandan parents’ experience. Lancet.

[9] Qazi SA (2013). An innovative multipartner research program to address detection, assessment and treatment of neonatal infections in low-resource settings. Pediatr Infect Dis J.

[10] Zaidi AK (2013). Scientific rationale for study design of community-based simplified antibiotic therapy trials in newborns and young infants with clinically diagnosed severe infections or fast breathing in South Asia and sub-Saharan Africa. Pediatr Infect Dis J.

[11] Baqui AH (2015). Safety and efficacy of alternative antibiotic regimens compared with 7 day injectable procaine benzylpenicillin and gentamicin for outpatient treatment of neonates and young infants with clinical signs of severe infection when referral is not possible: a randomised, open-label, equivalence trial. Lancet Global Health.

[12] Mir F (2017). Simplified antibiotic regimens for treatment of clinical severe infection in the outpatient setting when referral is not possible for young infants in Pakistan (Simplified Antibiotic Therapy Trial [SATT]): a randomised, open-label, equivalence trial. Lancet Global Health.

[13] Tshefu A (2015). Simplified antibiotic regimens compared with injectable procaine benzylpenicillin plus gentamicin for treatment of neonates and young infants with clinical signs of possible serious bacterial infection when referral is not possible: a randomised, open-label, equivalence trial. Lancet.

[14] World Health Organization, Guideline: Managing possible serious bacterial infection in young infants when referral is not feasible. Geneva: World Health Organization; 2015. 26447263

[15] National Technical Working Committee on Newborn Health, Bangladesh National Guideline (2015). Management of infection of the 0-59 days infants at union level facilities and NGO clinics without indoor facilities.

[16] Seddiky MA, Rahman ST (2015). Role of Union Health and Family Welfare Center (UH&FWC) to promote maternal education and reduce child mortality rate in Bangladesh. J Emerg Trends Educ Res Policy Stud.

[17] Ministry of Health and Family Welfare Bangladesh. Health Bulletin 2016. 2016 [Accessed 2 Feb 2017]; Available from: http://www.dghs.gov.bd/images/docs/Publicaations/HB%202016%20_2nd_edition_13_01_17.pdf.

[18] Baqui AH (2008). Effect of community-based newborn-care intervention package implemented through two service-delivery strategies in Sylhet district, Bangladesh: a cluster-randomised controlled trial. Lancet.

[19] MCHIP. MaMoni Health Systems Strenghtening Project-Bangladesh. [Accessed 6 Apr 2017]; Available from: http://www.mchip.net/node/89.

[20] World Health Organization. Community clinics in Bangladesh: Bringing health care to the doorstep of rural people. 2017 [Accessed 2 Feb 2017]; Available from: http://www.searo.who.int/mediacentre/events/community-clinic-bangladesh-story.pdf?ua=1.

[21] World Health Organization, Service availability and readiness assessment (SARA), in Implementation Guide, version 2.2. Geneva: World Health Organization; 2015.

[22] Baqui AH (2011). Levels, timing, and etiology of stillbirths in Sylhet district of Bangladesh. BMC Pregnancy Childbirth.

[23] Baqui AH (2006). Rates, timing and causes of neonatal deaths in rural India: implications for neonatal health programmes. Bull World Health Organ.

[24] Mitra DK (2016). Implementation of the ANISA protocol in Sylhet, Bangladesh: Challenges and Solutions. Pediatr Infect Dis J.

[25] Brassard M, Ritter D. The memory Jogger II: A Pocketguide of Tools for Continuous Improvement & Effective Planning. Goal/QPC; 1988.

[26] Peters HD, Tran TN, Adam T. Implementation research in health: a practical guide. Geneva: Alliance for Health Policy and Systems Research, World Health Organization; 2013.

[27] StataCorp, Stata Statistical Software: Release 14. 2015, StataCorp LP: College Station, TX.

[28] Ritchie J, Spencer L, Bryman A, Burgess R (1994). Qualitative data analysis for applied policy research, in Qualitative data analysis for applied policy research.

[29] Peters DH (2013). Implementation research: what it is and how to do it. BMJ.

[30] Habicht JP, Victora CG, Vaughan JP (1999). Evaluation designs for adequacy, plausibility and probability of public health programme performance and impact. Int J Epidemiol.

[31] Peters DH (2014). The application of systems thinking in health: why use systems thinking?. Health Res Policy Syst.

[32] Victora CG, Habicht JP, Bryce J (2004). Evidence-based public health: moving beyond randomized trials. Am J Public Health.

[33] Creswell J, Clark VP. Designing and Conducting Mixed Methods Research. 2nd Edition ed. 2011. USA: SAGE Publication Inc.

[34] Teddlie C. Tashakkori A. Foundations of mixed methods research: Integrating quantitative and qualitative approaches in the social and behavioral sciences. London: Sage; 2009.

[35] Proctor E (2011). Outcomes for implementation research: conceptual distinctions, measurement challenges, and research agenda. Adm Policy Ment Health.

